# Seafood Labeling in Croatia: Molecular Evidence and Regulatory Insights

**DOI:** 10.3390/foods14060917

**Published:** 2025-03-07

**Authors:** Dorotea Grbin, Snježana Zrnčić, Dražen Oraić, Matea Alfier, Mario Cindrić, Lucija Jović, Ivica Sučec, Ivana Giovanna Zupičić

**Affiliations:** 1 Croatian Veterinary Institute, Savska cesta 143, 10000 Zagreb, Croatia; grbin@veinst.hr (D.G.); oraic@veinst.hr (D.O.); alfier@veinst.hr (M.A.); zupicic@veinst.hr (I.G.Z.); 2 Institute Ruđer Bošković, Planinska 1, 10000 Zagreb, Croatia; mcindric@irb.hr; 3 Ministry of Agriculture, Forestry and Fisheries, Alexandera von Humboldta 4b, 10000 Zagreb, Croatia; lucija.jovic@mps.hr (L.J.); ivica.sucec@mps.hr (I.S.)

**Keywords:** DNA barcoding, seafood traceability, food fraud, mislabeling

## Abstract

Fisheries and aquaculture play a crucial role in global food security, yet species mislabeling remains a persistent challenge, undermining consumer trust and market transparency. Proper food labeling is essential for protecting public health due to the presence of unknown toxic or allergenic substances and preventing illegally sourced products from entering the market. Despite extensive research across Europe, seafood mislabeling in Croatia has remained unexplored. This study aims to provide the first comprehensive assessment of seafood labeling accuracy in Croatia, where fisheries are integral to the coastal economies and tourism. Using DNA barcoding of the COI gene, 109 seafood samples were collected over two years from various sources, including restaurants, markets, and fishing vessels, and analyzed for potential mislabeling. Results revealed a mislabeling rate of 3% among fish samples and 20% among cephalopods, with notable substitutions, such as the yellowfin tuna mislabeled as bigeye tuna and Bluefin tuna and the European squid mislabeled as Patagonian squid. Additionally, 38.5% of samples were partially labeled, while 32% lacked clear country-of-origin information, complicating traceability. While the findings align with the mislabeling rates in other European countries, this study underscores the ongoing challenges in seafood labeling compliance. Establishing standardized monitoring protocols will be essential for improving comparability and effectively addressing seafood fraud.

## 1. Introduction

Fisheries and aquaculture are vital to global food and nutrition security. In 2022, global production from these aquatic resources reached a record of 223.2 million tons, with aquaculture surpassing capture fisheries for the first time [1]. In Croatia, fisheries have historically sustained the coastal and island communities by providing year-round income and supporting the growing coastal tourism industry. The sector encompasses both small-scale and industrial fishing, with key target species such as Atlantic Bluefin tuna (*Thunnus thynnus*) and sardines (*Sardina pilchardus*). Additionally, the Adriatic squid (*Loligo vulgaris*) is a highly valued fishery catch, alongside various demersal fish mostly represented by species of the family Merlucciidae [2]. However, challenges persist, particularly illegal, unreported, and unregulated (IUU) fishing, as well as the inconsistencies in seafood labeling and traceability; these remain areas of concern [3], similar to many other countries worldwide facing these issues. Despite adherence to the European Union regulations, Croatia faces challenges in enforcing the seafood-labeling standards due to the gaps in traceability, especially for imported products. Additionally, the presence of partially labeled products, such as those generically marketed as “tuna” or “hake”, highlights the need for stricter enforcement of labeling requirements.

These limitations underscore the need for improved monitoring systems, stronger regulatory oversight, and greater consumer awareness to reduce seafood mislabeling. Deliberate mislabeling and the substitution of high-value species with cheaper or more readily available ones constitute Economically Motivated Adulteration (EMA), a form of food fraud [4]. Food fraud is more likely to occur with expensive and hard-to-source species [5,6], sometimes leading to the inclusion of endangered or threatened species in the products marketed as “sustainable” [7,8,9]. Mislabeling can also result from confusion between scientific, commercial, and common names, both across different countries and within the same region. Proper food labeling is essential for multiple reasons: it ensures food safety, enables effective inventory management, prevents illegally sourced products from entering the market, enables consumers to make informed choices, and protects public health [10]. Mislabeled or substituted fish sold in markets, fisheries, and restaurants can pose serious health risks due to the presence of unknown toxic or allergenic substances [11,12].

To address these concerns, the European Regulation EU 1379/2013, Art. 35, mandates the appropriate marking or labeling of fisheries and aquaculture products [13]. This information includes the commercial designation of the species and its scientific name, the production method, the catching area, and the fishing gear used. More recently, in January 2024, the European Union adopted new traceability measures for seafood, requiring all the import documents to be provided in digital form starting from 2026 [14].

The main methods for verifying food authenticity are either protein and/or DNA sequence analysis. While protein-based techniques work well for testing fresh products, their effectiveness decreases when analyzing highly processed foods [15]. In such cases, DNA-based methods are more reliable and represent an almost universal methodology for food traceability. One such technique is DNA barcoding, which uses a short, standardized DNA segment to identify species. It was developed in 2003 by researchers at the University of Guelph in Canada [16]. DNA barcoding is based on the mitochondrial gene cytochrome c oxidase subunit 1 (COI) and is now recommended as a standard method for seafood traceability, as it can address the high diversity in species or even subspecies. Using this technique, Galimberti et al. [17] successfully differentiated all the fish species in their study, proving that DNA barcoding is highly effective in accurately identifying the species, including their eggs, larvae, fillets, and fins.

The European Parliamentary Research Service (2014) [18] reported an increase in the number of food categories identified as particularly susceptible to fraud, with seafood being the second most likely, after olive oil [19]. The rate of seafood mislabeling detected through COI gene barcoding varies significantly across regions and countries [20], consumption source [21,22], particular seafood products [6,23,24,25,26], and conservation status [27,28], underscoring the global challenge of seafood fraud.

Over the past decade, a decline in the mislabeling rates has been observed in the European seafood sector. A study by Mariani et al. [29], which analyzed high-quality DNA sequence data from 1563 samples across six countries, found that only 4.93% of the samples were mislabeled according to European law. The overall mislabeling rates for each country were as follows: 2.7% (France), 3.25% (UK), 3.9% (Ireland), 6.21% (Germany), 6.7% (Portugal), and 8.9% (Spain). Research shows that seafood mislabeling rates are declining globally, although they remain high in non-European nations with legal systems differing from the EU’s [30]. Studies conducted between 2004 and 2016 highlight the persistence of this issue. For example, a survey of red snapper labeling in the US market using DNA barcoding found a mislabeling rate of 75% [31]. Similarly, a study by Oceana, the world’s largest ocean conservation organization, revealed that 33% of 1215 seafood samples from 674 US retail establishments were incorrectly labeled between 2010 and 2012 [32]. Additionally, a 2016 Oceana report reviewing over 200 studies across 55 countries found an average mislabeling rate of 20% in the global retail and catering sectors [33]. Notably high mislabeling rates were also observed in India (22% [34]), Thailand (24.44% [35]), Texas, USA (20.6% [36]), Brazil (24% [37]), Mexico (30.5% [38]), and South Africa (31% [39]).

Although Croatia’s fishing industry is significant due to the country’s extensive maritime resources, traditions, and its role as part of the Adriatic region, no comprehensive investigation has been carried out to identify the level of seafood mislabeling so far. As an EU member, Croatia must comply with stringent food labeling regulations, and investigating mislabeling helps ensure alignment with the EU standards. Given the mentioned challenges and the importance of accurate species identification for regulatory compliance, food safety, and consumer confidence, we conducted a barcoding study on seafood in Croatia with the support of the Ministry of Agriculture, Forestry, and Fisheries, Directorate of Fisheries. Our aim was to use DNA barcoding techniques to assess the accuracy of seafood labeling in all niches of the Croatian market, identify potential cases of mislabeling or fraud, and contribute to improving the traceability of seafood in Croatia.

## 2. Materials and Methods

### 2.1. Sampling

Fisheries Inspection Sector collected samples to verify seafood authenticity and ensure correct labeling in the marketing of fisheries and aquaculture products. This was done as a part of the program supported by the Ministry of Agriculture, Forestry, and Fisheries in compliance with European regulations and the Croatian Fisheries Act, ensuring a legally standardized approach. To obtain a pilot overview of seafood mislabeling in Croatia, samples were randomly collected across nine Croatian counties from diverse sources, including restaurants, fish markets, and retail chains. This randomized sampling strategy aimed to capture a broad spectrum of seafood products available to consumers, reflecting real-market conditions and providing insights into potential labeling inconsistencies across different points in the supply chain. Fisheries inspectors collected a tissue sample ranging from five to ten millimeters in length and 0.70–0.97 g in weight. To preserve the tissue, it was placed in a plastic polypropylene vial filled with 96% ethanol, ensuring full immersion for proper preservation. The vial was then tightly sealed with a screw cap and placed in a labeled protective bag for transport. Samples were delivered to our laboratory via courier service.

### 2.2. Ethics Approval

The Fisheries Inspectorate of Ministry of Agriculture, Forestry, and Fisheries submitted samples for barcoding. Since the animals were not used for experiments but for marketing purposes, their sacrifice followed the principles of good veterinary practice and adhered fully to animal welfare regulations, as outlined in the Act on Animal Protection in Research Purposes (Chapter 1, Article 5, Fish, Annex IV, Table 3) [40]. The Ethics Committee of the Croatian Veterinary Institute determined that no formal approval was required, and the study complied with national legislation.

### 2.3. DNA Extraction from Tissue and Sequencing Identification

DNA was extracted from approximately 25 mg of tissue using the innuPREP AniPath DNA/RNA Kit—IPC16 (Analytik Jena, Jena, Germany) on the InnuPure C16 touch (Analytik Jena, Jena, Germany), following the manufacturer’s instructions. The extracted DNA was stored at −20 °C and processed within one week of collection. For finfish samples, a ∼650-bp fragment of the mitochondrial cytochrome oxidase subunit I (COI) gene was amplified using universal primers, as described by Ward et al. [11]. For cephalopod samples, a ∼710-bp fragment of the COI gene was amplified with universal primers following the protocol by Folmer et al. [41] (Table 1).

We amplified the mitochondrial cytochrome oxidase subunit I (COI) gene using two sets of primers—FishF1, FishR1, and FishF2, FishR2 for finfish barcoding, and HCO and LCO primers for cephalopod barcoding (Table 1). All reactions were carried out with 2 µL of extracted DNA in a total volume of 25 µL using GoTaq G2 Hot Start Colorless Master Mix (Promega, Madison, WI, USA) on the ProFlex PCR System (Applied Biosystems, Waltham, MA, USA). The temperature profile for finfish barcoding was as follows: 2 min of denaturation at 95 °C, followed by 35 cycles of 94 °C for 30 s, annealing at 54 °C for 30 s, and extension at 72 °C for 1 min. A final extension was performed at 72 °C for 10 min, and the amplicons were stored at 4 °C. For cephalopod barcoding, the temperature profile was as follows: 4 min of denaturation at 95 °C, 35 cycles of 94 °C for 30 s, annealing at 52 °C for 1 min, and extension at 72 °C for 1 min. The final extension was carried out at 72 °C for 8 min, and the amplicons were stored at 4 °C. To visualize the amplified product, electrophoresis was performed on the QIAxcel Advanced System (Qiagen, Hilden, Germany) using the QIAxcel DNA Screening Kit. DNA samples that exhibited a positive amplification signal were submitted for sequencing to Macrogen Europe (Amsterdam, The Netherlands).

### 2.4. Mislabeling Determination

The obtained sequences were identified using the BLAST service of GenBank’s genomic databases and the Barcode of Life Data System (BOLD, v.4) [42,43], respectively. When the search returned a single species hit with ˃98% identity, this was considered positive species-level identification. A sample was declared mislabeled if the molecular identification did not match the taxonomic name of the seafood declared on the product.

### 2.5. Statistical Analysis

Descriptive statistical methods were applied to summarize the findings of this study. The proportions of different seafood species, labeling accuracy, and country of origin were calculated as percentages. Confidence intervals (CIs) were determined at a 95% confidence level for mislabeling rates and other key proportions to provide a measure of statistical reliability.

## 3. Results

Over a two-year period, from 2023 to 2024, a total of 109 samples were collected; they were obtained from restaurants (17.4%, n = 19), fish markets (22%, n = 24), retail chains (38.5%, n = 42), and fishing vessels (4.6%, n = 5). A total of 49 specimens (45%) were fresh, while 41 specimens (37.6%) were frozen at the time of sampling. The traceability of 19 samples (17.4%) was not possible; therefore, we were not able to assign these samples according to the sampling source and processing level.

**Figure 1:**
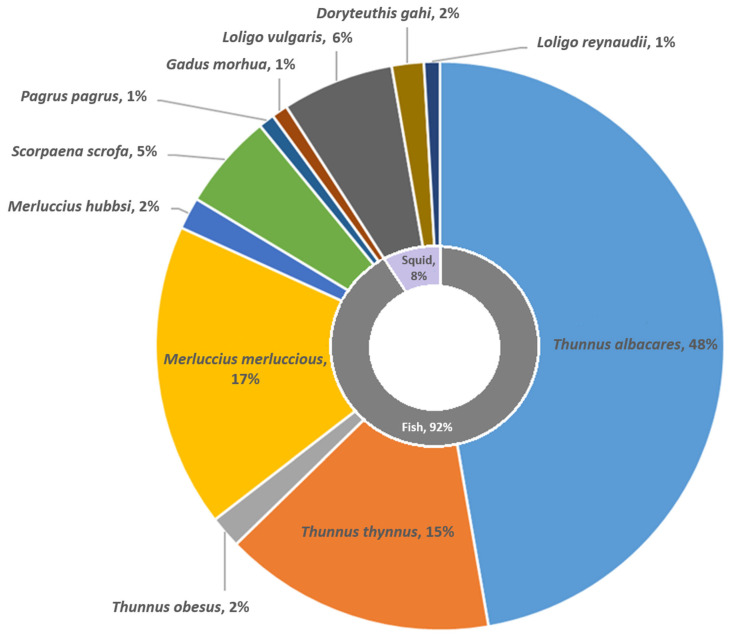
The distribution of collected specimens, identified at the species level using BLAST similarity searches.

**Figure 2:**
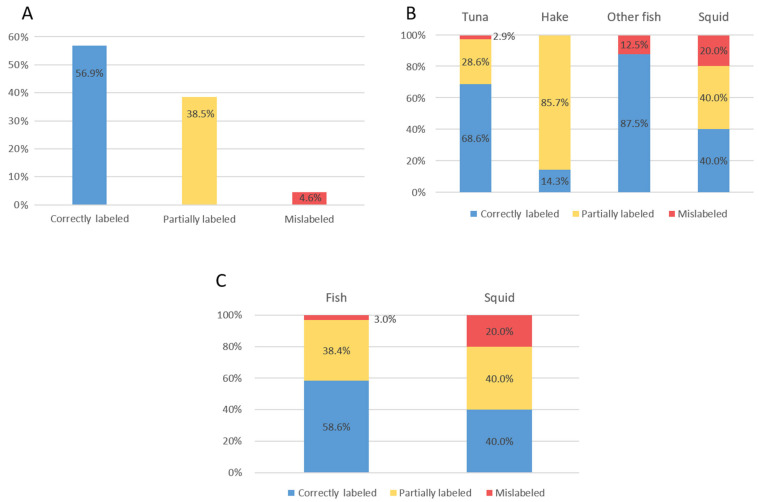
Seafood labeling accuracy: (**A**)—Overall labeling accuracy; (**B**)—Labeling accuracy between fish and squid samples; (**C**)—Labeling accuracy between specific groups of fish and squid.

The COI genes were successfully sequenced using two different primer sets for the 109 finfish and cephalopod commercial seafood products. All samples were identified at the species level with 98.06–100% nucleotide identity using both BLAST and BOLD databases, as shown in Tables S1–S3. The majority of samples were finfish (92%), while only 8% were cephalopods, all of which were squid species, either European squid (*Loligo vulgaris*), Patagonian longfin squid (*Loligo gahi*), or Cape Hope squid (*Loligo reynaudii*). Among the finfish samples, 65% were assigned to the genus *Thunnus*, 19% to the genus *Merluccius*, and the remaining 7% included other finfish species (Figure 1). We found that a relatively high number of samples, exactly 42 out of 109 (38.5%, CI [2.4%, 47.7%], with a confidence level of 95%), were only partially labeled (Figure 2A). Among the partially labeled samples, 63.6% contained only a scientific name, 9.1% contained a common trade name, while 9 tuna samples (27.3%) had a general “tuna filet” designation that did not include a precise description of a particular species. As shown in Figure 2B, hake samples exhibited the highest proportion of partial labels (86%), with many indicating *“Merluccius merluccius”* or *“Merluccius hubbsi”*.

**Figure 3:**
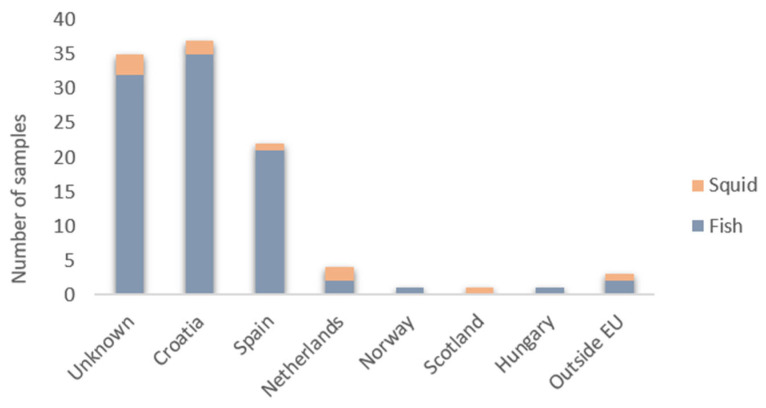
Countries of origin for the collected samples.

Based on the results of our sequencing and database search analysis, we found that five samples (4.6%, CI [0.66%, 8.51%]) were mislabeled (Figure 2A), with a mislabeling rate of 3% among finfish samples and 20% among squid samples (Figure 2C). Specifically, two samples of Yellowfin tuna (*Thunnus albacares*) were actually identified as Bigeye tuna (*Thunnus obesus*) and Bluefin tuna (*Thunnus thynnus*). Additionally, two samples labeled as European squid were found to be Patagonian squid and Cape Hope squid, while one sample of Red porgy (*Pagrus pagrus*) was misidentified as Atlantic cod (*Gadus morhua*). Two misidentified samples (Patagonian longfin squid and Bigeye tuna) were collected from restaurants, one misidentified tuna (Bluefin tuna) was obtained from a fish market, and for two misidentified samples (Red porgy and Cape Hope squid), the source of sampling was not documented. Additionally, for three out of five mislabeled samples (60%), there was no traceability (the producer was unknown), and the sampling source was not labeled. Our study revealed that 34% of the samples were imported (primarily from Spain, accounting for 20% of the total), while another 34% were sourced domestically from Croatia. A concerning proportion of samples (n = 35; 32%) had an unknown country of origin (Figure 3).

## 4. Discussion

To our knowledge, this study is the first large-scale survey assessing the extent of seafood mislabeling in Croatia. The growing global demand for fish and seafood has posed significant challenges to ensuring the product’s integrity and traceability. As the market expands, mislabeling has become increasingly prevalent, undermining consumer trust and raising concerns about sustainability, food safety, and fair-trade practices.

Before the implementation of European Regulation in 2013, mislabeling was notably high across Europe. For instance, Asensio et al. [44] reported that 83% of the commercial grouper products from markets throughout Madrid, Spain, were mislabeled. Similarly, Miller and Mariani [45] found that 25% of the randomly sampled cod and haddock from supermarkets, fishmongers, and take-away restaurants in Dublin, Ireland, were mislabeled, with smoked fish products exhibiting an even higher mislabeling rate of 82.4%. Filonzi et al. [46] analyzed the processed fish products in Italian markets and found that 32% were mislabeled, with 26% classified as serious fraud carrying significant economic and nutritional implications.

Despite a global average mislabeling rate of 30%, determined through a meta-analysis of 4500 seafood products across 51 publications [47], a general decreasing trend in fish species substitutions has been observed in Europe over the past decade. Recent research highlights a significant reduction in seafood mislabeling in Italy, where rates have declined from 32% to 11.6% over the past decade, and major fraud cases have decreased from 26.1% to 5.8% [48]. Minoudi et al. [49] detected a relatively low overall mislabeling (12.9%) in the Greek market during 2015–2018, compared to the study by Pazartzi et al. [50], where it exceeded 35%. These improvements are attributed to stringent regulations requiring detailed labeling, including common and scientific names as well as geographical regions of origin, as defined by the Food and Agriculture Organization (FAO) of the United Nations. Similarly, based on our current pilot analysis of 109 fish and squid commercial seafood products, Croatia has a low total mislabeling rate (4.6%). This low level of mislabeling is consistent with a study conducted in France, which analyzed 371 samples and found an overall mislabeling rate of 3.7% across the following five species: bluefin tuna, cod, yellowfin tuna, sole, and seabream [51]. These low mislabeling rates align with results from a large-scale study by Mariani et al. [29], which examined 1563 samples across six countries and found the overall mislabeling rates between 2.7 and 8.9%.

While these findings indicate significant improvements in the EU’s fish retail industry, comparing mislabeling levels across different studies remains challenging due to the methodology and scope. For example, a study by Paolacci et al. [52] highlighted significant differences in compliance with the legislation (*p* < 0.01), showing that unprocessed, non-prepacked products had a lower level of compliance (76%) compared to prepacked products (~96%). Nagalakshmi et al. [34] reported that among 22% of the mislabeled seafood products in India, mislabeling was most prevalent in the ready-to-eat products (28%), followed by ready-to-cook (18%), fresh (17%), and frozen products (7%). The same study found that restaurants were the primary source of seafood mislabeling (32%), followed by local markets (13%) and supermarkets (9%). Other researchers [53] have reported similar trends, with restaurants exhibiting a significantly higher incidence of mislabeling (61%) compared to supermarkets.

Potential biases may exist due to the non-systematic sample selection process, as the absence of a predefined protocol may have led to over- or under-representation of certain seafood types, supply chain stages, species, conservation statuses, product types, or geographic regions. Since samples were collected randomly by fishery inspectors, this lack of structured sampling may have introduced biases. However, the diversity of sources was intended to capture real market conditions and provide valuable insights into mislabeling practices to help guide future research and policy directions. Due to the low abundance of mislabeled species (n = 5) in our research, it was not possible to statistically identify the primary sources of mislabeling. However, it is noteworthy that in 2 out of 5 cases, proper traceability was lacking (e.g., the producer was unknown), underscoring that partial labeling still poses challenges to consumer trust and traceability. While partial labeling may offer some degree of transparency by providing scientific or trade names, it often omits key information—such as country of origin—creating ambiguity. This lack of clarity complicates the traceability efforts, undermines the consumers’ ability to make informed purchasing decisions, and contributes to regulatory non-compliance.

Mislabeling can occur at any stage of the product supply chain, especially since tracking the origin of samples is challenging. Distribution traceability is crucial for several reasons. Seafood pricing in global markets is influenced by species availability; however, this balance is disrupted by the improper introduction of species and illegal catches sold without market regulation and traceability [39]. Fraudulent substitutions deceive consumers, leading to resource scarcity, higher profits for unscrupulous sellers, and weak legislation that encourages mislabeling, posing health risks. Fish allergy represents the third most prominent cause of food allergies in children, after milk and eggs [54]. Hake is thought to have the strongest allergic reaction among the species that are frequently consumed in Europe [55], followed by cod, salmon, and pollack. In contrast, other species, such as tuna and halibut, are less likely to cause allergies [56,57]. More than 20% mislabeling has been detected in the market lots of hake in Spain [58]. Hake has previously been reported to have high mislabeling rates, such as 11.1% in Portugal [29], 43% in Spain [59], and up to 50% in Germany [60]. In this research, we pinpointed that 85.7% of the hake samples were partially labeled. However, we did not record major fraud among hake species but among squid and tuna species.

Recently, cephalopod product substitution was confirmed in Greece [61], with mislabeling rates reaching 40.4%. European squid, known for its superior taste and texture due to high amino acid and low glycogen content, commands a higher market price [62]. Two mislabeled squid samples in our study, falsely labeled as European squid, likely represent intentional mislabeling motivated by economic incentives tied to the higher market value of this species.

Regarding the mislabeled tuna specimens, Yellowfin tuna identified as Bigeye tuna is an evident food fraud incident, attributed to a lack of proper traceability mechanisms or misidentification during processing. On the contrary, the mislabeling of Bluefin tuna could potentially represent an incentive in order to facilitate market access for illegally landed seafood. This is because the Northeast Atlantic and Mediterranean bluefin tuna stocks were severely overexploited before recovery efforts that included reducing the total allowable catch (TAC) quotas; decreasing the fishing efforts; eliminating illegal, unreported, and unregulated (IUU) fishing; and ensuring compliance with quotas [63].

Another tuna-related issue we pinpoint is the labeling of fresh and frozen products with a generic name, such as “tuna”. Although current labeling legislation in the EU establishes the obligation to indicate commercial name and species in the case of fresh, frozen, smoked, and dried seafood products (EU1379/2013), we found nine products labeled as “tuna” or “tuna filet”. While the general term “tuna” is widely used by consumers, its ambiguity hinders market transparency and can negatively impact the proper management of tuna species stocks [64,65]. In this context, DNA barcoding serves as a valuable tool for species identification, particularly for those frequently misidentified or lacking proper labeling.

## 5. Conclusions

This first study on seafood mislabeling in Croatia analyzed 109 products using molecular techniques, revealing a 4.6% mislabeling rate consistent with the low levels in Europe; this indicates a potential progress in labeling accuracy. DNA barcoding has proven to be a valuable tool in seafood mislabeling analysis and should be used in further investigations, as mislabeling rates can vary significantly depending on the sampling methods, species examined, and geographical context. However, the seafood labeling accuracy using DNA barcoding should be complemented with the use of proteomic methods.

Special attention should be given to squid, as they are highly susceptible to mislabeling, as well as protected species, due to concerns about deliberate mislabeling as sustainable. Additionally, stricter oversight is needed for inadequately labeled products, particularly in restaurants and ready-to-eat markets, where mislabeling rates are higher.

To obtain statistically robust data, large-scale studies across various regions are needed for a more accurate assessment of seafood labeling. While this study offers the first comprehensive insight into mislabeling in Croatia, its limitations include random sampling without a predefined protocol and an over-representation of tuna, which may have influenced the results. Future research should adopt a more structured sampling approach for balanced sources (restaurants, fish markets, and retail chains) and species representation.

This research highlights Croatia’s mislabeling status, contributes to the European and global dialogue on seafood fraud, and advocates for stricter labeling practices to ensure transparency, sustainability, and regulatory compliance, while emphasizing the need for standardized traceability approaches to improve comparability and better understand seafood fraud trends.

## Figures and Tables

**Table 1:** Set of primers used in this study.

Primer	Sequence 5′-3′	Reference
FishF1	TCAACCAACCACAAAGACATTGGCAC	Ward et al., (2009) [11]
FishF2	TCGACTAATCATAAAGATATCGGCAC	Ward et al., (2009) [11]
FishR1	TAGACTTCTGGGTGGCCAAAGAATCA	Ward et al., (2009) [11]
FishR2	ACTTCAGGGTGACCGAAGAATCAGAA	Ward et al., (2009) [11]
LCO1490	GGTCAACAAATCATAAAGATATTGG	Folmer et al., (1994) [41]
HC02198	TAAACTTCAGGGTGACCAAAAAATCA	Folmer et al., (1994) [41]

## Data Availability

The original contributions presented in this study are included in the article/Supplementary Materials. Further inquiries can be directed to the corresponding author.

## Supplementary Materials

The following supporting information can be downloaded at https://www.mdpi.com/article/10.3390/foods14060917/s1: Tables S1–S3 consist of data on samples obtained from Croatian market. The table shows declared name (Croatian name; scientific name), the species identified by either BLAST (https://www.ncbi.nlm.nih.gov/BLAST, accessed on 15 December 2024) or BOLD (https://v3.boldsystems.org/, accessed on 28 February 2025.), the similarity between the amplified and the most similar sequence existing in GenBank or BOLD (%) databases for two sets of primers used in the study, and labeling status marked as—correctly labeled/partially labeled/mislabeled.
